# Ethyl 2-(6-amino-5-cyano-3,4-dimethyl-2*H*,4*H*-pyrano[2,3-*c*]pyrazol-4-yl)acetate

**DOI:** 10.1107/S1600536810015540

**Published:** 2010-04-30

**Authors:** M. Kannan, Kandhasamy Kumaravel, Gnanasambandam Vasuki, R. Krishna

**Affiliations:** a Centre for Bioinformatics, Pondicherry University, Puducherry 605 014, India; b Department of chemistry, Pondicherry university, Puducherry 605 014, India

## Abstract

In he title compound, C_13_H_16_N_4_O_3_, the pyrazole ring is planar (r.m.s. deviation = 0.054 Å). The pyran ring is not planar; the mean plane makes a dihedral angle of 1.9 (1)° with the pyrazole ring. In the crystal structure, inter­molecular N—H⋯N and N—H⋯O inter­actions lead to a linear chain motif.

## Related literature

For biological applications of pyrazole and pyran­opyrazole derivatives, see: Wamhoff *et al.* (1993 ▶).; Velaparthi *et al.* (2008 ▶); Magedov *et al.* (2007 ▶); Rovnyak *et al.* (1982 ▶). For the synthesis, see: Vasuki & Kumaravel (2008 ▶).
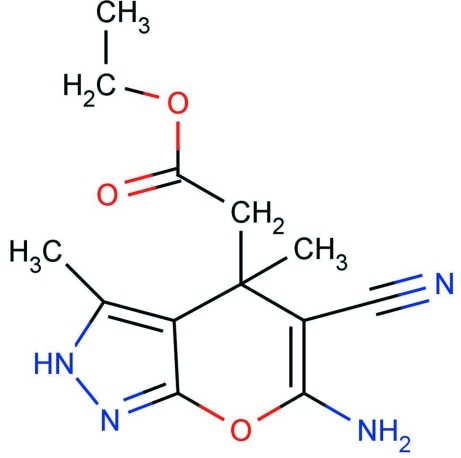


## Experimental

### 

#### Crystal data

C_13_H_16_N_4_O_3_
*M* *_r_* = 276.30
Triclinic, 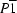

*a* = 6.961 (5) Å
*b* = 7.373 (5) Å
*c* = 14.535 (5) Å
α = 86.405 (5)°
β = 85.183 (5)°
γ = 65.726 (5)°
*V* = 677.3 (7) Å^3^
*Z* = 2
Mo *K*α radiation
μ = 0.10 mm^−1^
*T* = 293 K
0.25 × 0.20 × 0.20 mm

#### Data collection

Bruker Kappa APEXII CCD diffractometer
Absorption correction: multi-scan (*SADABS*; Bruker 2004 ▶) *T*_min_ = 0.976, *T*_max_ = 0.981
12811 measured reflections
2385 independent reflections
2130 reflections with *I* > 2σ(*I*)
*R*_int_ = 0.020

#### Refinement

*R*[*F* ^2^ > 2σ(*F* ^2^)] = 0.041
*wR*(*F* ^2^) = 0.117
*S* = 1.02
2378 reflections
184 parameters
H-atom parameters constrained
Δρ_max_ = 0.30 e Å^−3^
Δρ_min_ = −0.26 e Å^−3^

### 

Data collection: *APEX2* (Bruker, 2004 ▶); cell refinement: *APEX2* and *SAINT* (Bruker, 2004 ▶); data reduction: *SAINT* and *XPREP* (Bruker, 2004 ▶); program(s) used to solve structure: *SHELXS97* (Sheldrick, 2008 ▶); program(s) used to refine structure: *SHELXL97* (Sheldrick, 2008 ▶); molecular graphics: *ORTEP-3 for Windows* (Farrugia, 1997 ▶); software used to prepare material for publication: *PLATON* (Spek, 2009 ▶).

## Supplementary Material

Crystal structure: contains datablocks I, global. DOI: 10.1107/S1600536810015540/ng2761sup1.cif

Structure factors: contains datablocks I. DOI: 10.1107/S1600536810015540/ng2761Isup2.hkl

Additional supplementary materials:  crystallographic information; 3D view; checkCIF report

## Figures and Tables

**Table 1:** Hydrogen-bond geometry (Å, °)

*D*—H⋯*A*	*D*—H	H⋯*A*	*D*⋯*A*	*D*—H⋯*A*
N2—H2⋯O2^i^	0.86	2.55	3.274 (2)	142
N2—H2⋯N1^ii^	0.86	2.37	2.956 (2)	126
N3—H3*A*⋯N4^iii^	0.86	2.19	3.012 (2)	160
N3—H3*B*⋯O3^iv^	0.86	2.40	3.192 (2)	154

Symmetry codes: (i) 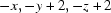; (ii) 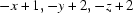; (iii) 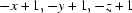; (iv) 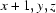.

**Fig. 1.**
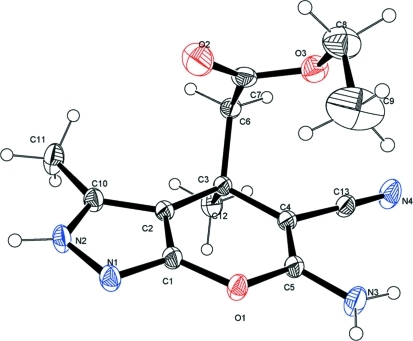
: The molecular structure of (I), showing the atom-numbering scheme and displacement ellipsoids drawn at the 30% probability level.

**Fig. 2.**
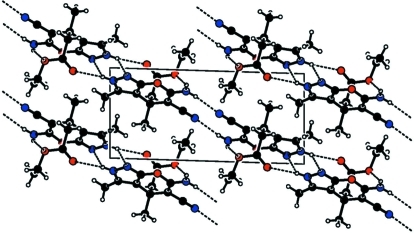
: The crystal packing of (I), showing intermolecular hydrogen bonding interactions as dashed lines.

## supplementary crystallographic information

### Comment

Pyrazole, Pyranopyrazoles and its derivates possess antimicrobial (Velaparthi *et al.*,2008),anticancer (Magedov *et al.*,2007) and anti-inflammatory (Rovnyak *et al.*,1982) properties,which made them to be used as a medicines and as biodegradable agrochemiclas (Wamhoff *et al.*, 1993).Wide variety of biological importances of these molecules made the quest for their crystal study and accordingly we have synthesized the title compound by multi-component reaction which compiles with the principles of green chemistry and reported the crystal structure of the title compound.

The compound was crystallized by slow evaporation technique using ethanol as solvent at room temperature. The title compound, (I) was centrosymmetric and it has triclinic crystal system with the space group of P-1. The pyrazole groups are essentially planar, with a mean deviation of 0.0542 Å from the least square plane defined by the five atoms (N1 to C1). The pyran ring deviates significantly from the plane and it has dihedral angle of 1.93 (0.06)° with pyrazole ring. The ethyl acetate group has dihedral angle of 49.99 (0.07)° with pyran ring to which it is attached (Fig. 1). The intermolecular hydrogen bond was formed between N2···N1, N3···N4, N2—H···O2 and N3—H···O3 with distance of 2.956 (2), 3.012 (2), 3.274 (2) and 3.192 (2) Å respectively (Table.1). These intermolecular interactions help in theformation three-dimensional network and crystal packing (Fig. 2) of (I).

### Experimental

The titled compound was prepared bythe successive addition of malononitrile (0.132 g, 2 mmol) and piperidine (5 mol%) to a stirred aqueous mixture of hydrazine hydrate 96% 1 (0.107 g, 2 mmol)and ethyl acetoacetate 2 (0.520 g, 4 mmol) at room temperature under an openatmosphere with vigorous stirring for 5–10 min. The precipitated solid wasfiltered, washed with water and then with a mixture of ethyl acetate/hexane(20:80) (Vasuki & Kumaravel, 2008). The product obtained was pure by TLCand ^1^H NMR spectroscopy. However, the products were further purifiedby recrystallization from ethanol. Analysis calculated for ethyl2-(6-amino-5-cyano-3,4-dimethyl-2,4-dihydropyrano[2,3-c]pyrazol-4-yl) acetate showed that it has C_13_, H_16_,*N*_4_, O_3_.

### Refinement

The non-hydrogen atoms where refined anisotropically whereas hydrogen atoms were refined isotropically. The H atoms were geometrically placed (N—H = 0.86 Å, and C—H=0.93-0.97 Å) and refined as riding with U_iso_(H) = 1.2-1.5 U_eq_ (parent atom).

### Crystal data

**Table:**

C_13_H_16_N_4_O_3_	*Z* = 2
*M**_r_* = 276.30	*F*(000) = 292
Triclinic, *P*1	*D*_x_ = 1.355 Mg m^−^^3^
Hall symbol: -P 1	Mo *K*α radiation, λ = 0.71069 Å
*a* = 6.961 (5) Å	Cell parameters from 7682 reflections
*b* = 7.373 (5) Å	θ = 2.8–32.8°
*c* = 14.535 (5) Å	µ = 0.10 mm^−^^1^
α = 86.405 (5)°	*T* = 293 K
β = 85.183 (5)°	Block, colourless
γ = 65.726 (5)°	0.25 × 0.20 × 0.20 mm
*V* = 677.3 (7) Å^3^	

### Data collection

**Table:**

Bruker Kappa APEXII CCD diffractometer	2385 independent reflections
Radiation source: fine-focus sealed tube	2130 reflections with *I* > 2σ(*I*)
graphite	*R*_int_ = 0.020
Detector resolution: 0 pixels mm^-1^	θ_max_ = 25.0°, θ_min_ = 2.8°
ω and φ scan	*h* = −8→8
Absorption correction: multi-scan (*SADABS*; Bruker 2004)	*k* = −8→8
*T*_min_ = 0.976, *T*_max_ = 0.981	*l* = −17→17
12811 measured reflections	

### Refinement

**Table:**

Refinement on *F*^2^	Primary atom site location: structure-invariant direct methods
Least-squares matrix: full	Secondary atom site location: difference Fourier map
*R*[*F*^2^ > 2σ(*F*^2^)] = 0.041	Hydrogen site location: inferred from neighbouring sites
*wR*(*F*^2^) = 0.117	H-atom parameters constrained
*S* = 1.02	*w* = 1/[σ^2^(*F*_o_^2^) + (0.0655*P*)^2^ + 0.2561*P*] where *P* = (*F*_o_^2^ + 2*F*_c_^2^)/3
2378 reflections	(Δ/σ)_max_ = 0.001
184 parameters	Δρ_max_ = 0.30 e Å^−^^3^
0 restraints	Δρ_min_ = −0.26 e Å^−^^3^

### Special details

**Table:**

Geometry. All esds (except the esd in the dihedral angle between two l.s. planes) are estimated using the full covariance matrix. The cell esds are taken into account individually in the estimation of esds in distances, angles and torsion angles; correlations between esds in cell parameters are only used when they are defined by crystal symmetry. An approximate (isotropic) treatment of cell esds is used for estimating esds involving l.s. planes.
Refinement. Refinement of *F*^2^ against ALL reflections. The weighted *R*-factor wR and goodness of fit *S* are based on *F*^2^, conventional *R*-factors *R* are based on *F*, with *F* set to zero for negative *F*^2^. The threshold expression of *F*^2^ > σ(*F*^2^) is used only for calculating *R*-factors(gt) etc. and is not relevant to the choice of reflections for refinement. *R*-factors based on *F*^2^ are statistically about twice as large as those based on *F*, and *R*- factors based on ALL data will be even larger.Some "bad" relections were omitted in the refinement.

### Fractional atomic coordinates and isotropic or equivalent isotropic displacement parameters (Å^2^)

**Table:**

	*x*	*y*	*z*	*U*_iso_*/*U*_eq_	
C1	0.4375 (2)	0.8266 (2)	0.84125 (10)	0.0288 (3)	
C2	0.3125 (2)	0.7231 (2)	0.86328 (9)	0.0271 (3)	
C3	0.2551 (2)	0.6151 (2)	0.79351 (9)	0.0271 (3)	
C4	0.3682 (2)	0.6404 (2)	0.70213 (9)	0.0282 (3)	
C5	0.4908 (2)	0.7439 (2)	0.68793 (10)	0.0306 (3)	
C6	0.0126 (2)	0.7009 (2)	0.78369 (10)	0.0329 (3)	
H6A	−0.0164	0.6276	0.7375	0.040*	
H6B	−0.0563	0.6818	0.8420	0.040*	
C7	−0.0780 (2)	0.9167 (2)	0.75667 (11)	0.0378 (4)	
C8	−0.1525 (3)	1.1488 (3)	0.62848 (17)	0.0654 (6)	
H8A	−0.2528	1.2402	0.6724	0.078*	
H8B	−0.2217	1.1636	0.5715	0.078*	
C9	0.0317 (5)	1.1990 (4)	0.6104 (3)	0.1088 (12)	
H9A	0.0887	1.2023	0.6679	0.163*	
H9B	−0.0109	1.3271	0.5794	0.163*	
H9C	0.1375	1.1003	0.5722	0.163*	
C10	0.2672 (2)	0.7477 (2)	0.95731 (10)	0.0320 (3)	
C11	0.1408 (3)	0.6754 (3)	1.02539 (11)	0.0487 (5)	
H11A	0.2177	0.5356	1.0380	0.073*	
H11B	0.0091	0.6978	1.0006	0.073*	
H11C	0.1140	0.7461	1.0816	0.073*	
C12	0.3282 (3)	0.3931 (2)	0.82003 (11)	0.0385 (4)	
H12A	0.4780	0.3351	0.8261	0.058*	
H12B	0.2951	0.3270	0.7729	0.058*	
H12C	0.2574	0.3782	0.8777	0.058*	
C13	0.3419 (2)	0.5481 (2)	0.62484 (10)	0.0354 (4)	
N1	0.4738 (2)	0.9108 (2)	0.91081 (8)	0.0351 (3)	
N2	0.3649 (2)	0.8596 (2)	0.98199 (8)	0.0357 (3)	
H2	0.3589	0.8954	1.0377	0.043*	
N3	0.5923 (2)	0.7662 (2)	0.60839 (9)	0.0474 (4)	
H3A	0.5831	0.7114	0.5594	0.057*	
H3B	0.6667	0.8352	0.6064	0.057*	
N4	0.3189 (3)	0.4715 (3)	0.56322 (10)	0.0555 (4)	
O1	0.52556 (17)	0.84545 (17)	0.75515 (7)	0.0367 (3)	
O2	−0.1338 (2)	1.0482 (2)	0.81014 (10)	0.0659 (4)	
O3	−0.08990 (18)	0.94621 (17)	0.66504 (8)	0.0450 (3)	

### Atomic displacement parameters (Å^2^)

**Table:**

	*U*^11^	*U*^22^	*U*^33^	*U*^12^	*U*^13^	*U*^23^
C1	0.0306 (7)	0.0354 (7)	0.0243 (7)	−0.0169 (6)	−0.0013 (5)	−0.0047 (6)
C2	0.0271 (7)	0.0322 (7)	0.0246 (7)	−0.0142 (6)	−0.0018 (5)	−0.0038 (5)
C3	0.0277 (7)	0.0322 (7)	0.0250 (7)	−0.0153 (6)	−0.0012 (5)	−0.0050 (5)
C4	0.0269 (7)	0.0353 (7)	0.0253 (7)	−0.0148 (6)	−0.0007 (5)	−0.0071 (6)
C5	0.0307 (7)	0.0399 (8)	0.0245 (7)	−0.0172 (6)	−0.0006 (6)	−0.0069 (6)
C6	0.0291 (7)	0.0450 (8)	0.0312 (8)	−0.0213 (6)	0.0005 (6)	−0.0068 (6)
C7	0.0250 (7)	0.0454 (9)	0.0434 (9)	−0.0128 (6)	−0.0036 (6)	−0.0124 (7)
C8	0.0592 (12)	0.0478 (11)	0.0810 (15)	−0.0144 (9)	−0.0116 (11)	0.0124 (10)
C9	0.096 (2)	0.0740 (17)	0.171 (3)	−0.0526 (16)	−0.029 (2)	0.0415 (19)
C10	0.0376 (8)	0.0365 (8)	0.0267 (7)	−0.0196 (6)	−0.0016 (6)	−0.0048 (6)
C11	0.0692 (12)	0.0637 (11)	0.0298 (8)	−0.0450 (10)	0.0083 (8)	−0.0090 (8)
C12	0.0481 (9)	0.0344 (8)	0.0364 (8)	−0.0197 (7)	−0.0038 (7)	−0.0028 (6)
C13	0.0362 (8)	0.0488 (9)	0.0289 (8)	−0.0255 (7)	0.0042 (6)	−0.0088 (7)
N1	0.0430 (7)	0.0457 (7)	0.0273 (6)	−0.0284 (6)	−0.0011 (5)	−0.0067 (5)
N2	0.0487 (8)	0.0459 (7)	0.0224 (6)	−0.0286 (6)	−0.0015 (5)	−0.0069 (5)
N3	0.0591 (9)	0.0738 (10)	0.0300 (7)	−0.0484 (8)	0.0114 (6)	−0.0162 (7)
N4	0.0675 (10)	0.0859 (12)	0.0357 (8)	−0.0534 (9)	0.0097 (7)	−0.0243 (8)
O1	0.0446 (6)	0.0537 (7)	0.0266 (6)	−0.0350 (5)	0.0047 (4)	−0.0106 (5)
O2	0.0652 (9)	0.0518 (8)	0.0677 (9)	−0.0048 (7)	−0.0160 (7)	−0.0270 (7)
O3	0.0440 (7)	0.0432 (6)	0.0457 (7)	−0.0163 (5)	−0.0008 (5)	−0.0005 (5)

### Geometric parameters (Å, °)

**Table:**

C1—N1	1.3125 (19)	C8—H8A	0.9700
C1—O1	1.3714 (18)	C8—H8B	0.9700
C1—C2	1.383 (2)	C9—H9A	0.9600
C2—C10	1.383 (2)	C9—H9B	0.9600
C2—C3	1.5006 (19)	C9—H9C	0.9600
C3—C4	1.527 (2)	C10—N2	1.346 (2)
C3—C12	1.535 (2)	C10—C11	1.487 (2)
C3—C6	1.556 (2)	C11—H11A	0.9600
C4—C5	1.356 (2)	C11—H11B	0.9600
C4—C13	1.411 (2)	C11—H11C	0.9600
C5—N3	1.341 (2)	C12—H12A	0.9600
C5—O1	1.3616 (17)	C12—H12B	0.9600
C6—C7	1.490 (2)	C12—H12C	0.9600
C6—H6A	0.9700	C13—N4	1.144 (2)
C6—H6B	0.9700	N1—N2	1.3572 (18)
C7—O2	1.195 (2)	N2—H2	0.8600
C7—O3	1.340 (2)	N3—H3A	0.8600
C8—O3	1.452 (2)	N3—H3B	0.8600
C8—C9	1.474 (4)		
			
N1—C1—O1	118.81 (13)	H8A—C8—H8B	108.0
N1—C1—C2	115.28 (13)	C8—C9—H9A	109.5
O1—C1—C2	125.90 (13)	C8—C9—H9B	109.5
C1—C2—C10	103.21 (13)	H9A—C9—H9B	109.5
C1—C2—C3	123.21 (13)	C8—C9—H9C	109.5
C10—C2—C3	133.58 (13)	H9A—C9—H9C	109.5
C2—C3—C4	105.90 (12)	H9B—C9—H9C	109.5
C2—C3—C12	111.39 (12)	N2—C10—C2	106.01 (13)
C4—C3—C12	109.91 (12)	N2—C10—C11	122.04 (14)
C2—C3—C6	112.12 (11)	C2—C10—C11	131.95 (14)
C4—C3—C6	110.22 (12)	C10—C11—H11A	109.5
C12—C3—C6	107.33 (12)	C10—C11—H11B	109.5
C5—C4—C13	116.78 (13)	H11A—C11—H11B	109.5
C5—C4—C3	126.29 (13)	C10—C11—H11C	109.5
C13—C4—C3	116.93 (13)	H11A—C11—H11C	109.5
N3—C5—C4	126.98 (14)	H11B—C11—H11C	109.5
N3—C5—O1	109.58 (13)	C3—C12—H12A	109.5
C4—C5—O1	123.44 (13)	C3—C12—H12B	109.5
C7—C6—C3	112.62 (12)	H12A—C12—H12B	109.5
C7—C6—H6A	109.1	C3—C12—H12C	109.5
C3—C6—H6A	109.1	H12A—C12—H12C	109.5
C7—C6—H6B	109.1	H12B—C12—H12C	109.5
C3—C6—H6B	109.1	N4—C13—C4	178.79 (17)
H6A—C6—H6B	107.8	C1—N1—N2	101.70 (12)
O2—C7—O3	123.82 (17)	C10—N2—N1	113.80 (12)
O2—C7—C6	124.23 (16)	C10—N2—H2	123.1
O3—C7—C6	111.94 (13)	N1—N2—H2	123.1
O3—C8—C9	111.08 (18)	C5—N3—H3A	120.0
O3—C8—H8A	109.4	C5—N3—H3B	120.0
C9—C8—H8A	109.4	H3A—N3—H3B	120.0
O3—C8—H8B	109.4	C5—O1—C1	115.14 (12)
C9—C8—H8B	109.4	C7—O3—C8	117.56 (15)
			
N1—C1—C2—C10	0.13 (17)	C12—C3—C6—C7	−179.24 (12)
O1—C1—C2—C10	179.38 (13)	C3—C6—C7—O2	−85.9 (2)
N1—C1—C2—C3	−179.36 (12)	C3—C6—C7—O3	94.18 (15)
O1—C1—C2—C3	−0.1 (2)	C1—C2—C10—N2	0.13 (16)
C1—C2—C3—C4	2.14 (18)	C3—C2—C10—N2	179.54 (14)
C10—C2—C3—C4	−177.17 (15)	C1—C2—C10—C11	−179.84 (17)
C1—C2—C3—C12	121.60 (15)	C3—C2—C10—C11	−0.4 (3)
C10—C2—C3—C12	−57.7 (2)	C5—C4—C13—N4	169 (9)
C1—C2—C3—C6	−118.10 (15)	C3—C4—C13—N4	−11 (9)
C10—C2—C3—C6	62.6 (2)	O1—C1—N1—N2	−179.63 (12)
C2—C3—C4—C5	−1.37 (19)	C2—C1—N1—N2	−0.32 (17)
C12—C3—C4—C5	−121.80 (16)	C2—C10—N2—N1	−0.35 (17)
C6—C3—C4—C5	120.10 (16)	C11—C10—N2—N1	179.63 (15)
C2—C3—C4—C13	178.97 (12)	C1—N1—N2—C10	0.41 (17)
C12—C3—C4—C13	58.54 (17)	N3—C5—O1—C1	−177.10 (12)
C6—C3—C4—C13	−59.56 (17)	C4—C5—O1—C1	3.8 (2)
C13—C4—C5—N3	−0.9 (2)	N1—C1—O1—C5	176.23 (12)
C3—C4—C5—N3	179.41 (14)	C2—C1—O1—C5	−3.0 (2)
C13—C4—C5—O1	178.03 (13)	O2—C7—O3—C8	7.0 (2)
C3—C4—C5—O1	−1.6 (2)	C6—C7—O3—C8	−173.07 (14)
C2—C3—C6—C7	58.13 (16)	C9—C8—O3—C7	87.2 (3)
C4—C3—C6—C7	−59.57 (16)		

### Hydrogen-bond geometry (Å, °)

**Table:**

*D*—H···*A*	*D*—H	H···*A*	*D*···*A*	*D*—H···*A*
N2—H2···O2^i^	0.86	2.55	3.274 (2)	142
N2—H2···N1^ii^	0.86	2.37	2.956 (2)	126
N3—H3A···N4^iii^	0.86	2.19	3.012 (2)	160
N3—H3B···O3^iv^	0.86	2.40	3.192 (2)	154

Symmetry codes: (i) −*x*, −*y*+2, −*z*+2; (ii) −*x*+1, −*y*+2, −*z*+2; (iii) −*x*+1, −*y*+1, −*z*+1; (iv) *x*+1, *y*, *z*.
